# Modeling the Chlorine Series from the Treatment Plant of Drinking Water in Constanta, Romania

**DOI:** 10.3390/toxics11080699

**Published:** 2023-08-13

**Authors:** Alina Bărbulescu, Lucica Barbeș

**Affiliations:** 1 Department of Civil Engineering, Transilvania University of Brașov, 5 Turnului Str., 500152 Brasov, Romania; alina.barbulescu@unitbv.ro; 2 Department of Chemistry and Chemical Engineering, Ovidius University of Constanța, 124 Mamaia Bd., 900152 Constanta, Romania; 3 Doctoral School of Biotechnical Systems Engineering, Politehnica University of Bucharest, 313, Splaiul Independentei, 060042 Bucharest, Romania

**Keywords:** free chlorine residual concentration series, modeling, forecast, water treatment plant

## Abstract

Ensuring good drinking water quality, which does not damage the population’s health, should be a priority of decision factors. Therefore, water treatment must be carried out to remove the contaminants. Chlorination is one of the most used treatment procedures. Modeling the free chlorine residual concentration series in the water distribution network provides the water supply managers with a tool for predicting residual chlorine concentration in the networks. With regard to this idea, this article proposes alternative models for the monthly free chlorine residual concentration series collected at the Palas Constanta Water Treatment Plant, in Romania, from January 2013 to December 2018. The forecasts based on the determined models are provided, and the best results are highlighted.

## 1. Introduction

Drinking water quality is essential, given its impact on the population’s health [1]. Therefore, ensuring a sufficient quantity and adequate quality must be a priority of each state/community to improve the health indicators and the population’s well-being [2]. The urban population’s primary drinking water supply sources are surface water and groundwater, whereas wells are used in rural areas [3]. In an ideal scenario, a water supply system would operate continuously, without changes in flow rate or other special conditions for individual treatment processes, when the raw water quality and quantity are constant. In reality, ideal conditions are not always met [4,5]. Given that various contaminants can affect the drinking water quality, it is crucial to treat the water before its distribution for consumption [6,7].

Due to its effectiveness (in killing viruses, bacteria, etc.), environmental feasibility, and long-lasting effects, chlorine is the primary disinfectant used for drinking water treatment [8,9]. Hypochlorous and hydrochloric acids are produced by adding chlorine or its derivatives to the raw water [10]. The active element in the disinfection process (the hypochlorite ion) results from the dissociation of the hypochlorous acid. During the water treatment, chlorine oxidizes the mineral substances and then produces chloramines by reacting with ammonia. Supplementing the chlorine dose leads to chloramine oxidation, increasing the free chlorine residual level [11,12], which is crucial for effective disinfection. The laboratory analyses performed on water samples taken at the outlet of the water treatment station and the distribution network indicate the disinfection stages and the necessary chlorine doses for ensuring water quality [13,14]. A balance in the chlorine dosing must be kept to protect the population against contamination, on the one hand, and avoid the by-products’ formation and pipes’ corrosion, on the other hand [14,15]. In these conditions, models that accurately predict the free chlorine residual in the distribution system have been proposed as a first step for optimizing the water treatment plant functioning.

Ghang et al. [16] introduced a chlorine decay model based on potential chlorine decay mechanisms and evaluated its performances on four raw surface and alum-treated waters. The results prove that the proposed model accurately predicts free chlorine residuals (R^2^ = 0.98). Gómez-Coronel et al. [17] reported satisfactory results in the chlorine concentration at the input of a water distribution system simulated in EPANET, with a genetic algorithm implemented in MATLAB. The EPANET MSX software was used to model chlorine decay in Algarve’s drinking water supply systems [18]. García-Ávila et al. [19] employed the same tool with a built-in first-order equation for modeling chlorine decay for a case study from Ecuador. Nejjari et al. [20] proposed a methodology for efficiently calibrating the free chlorine decay models tested on the Barcelona water transport network. Zhang et al. [21] elaborated a model for integrating water quality and operation for forecasting water production (using a genetic algorithm-enhanced artificial neural network). In contrast, other authors focused on optimizing the chlorine dosing [22,23].

Quantifying chlorine residual, turbidity, standard plate count (SPC), coliforms, etc., was performed using statistical methods in a water distribution system from Pakistan [24]. The correlations between the coliforms’ presence in the water and the free chlorine content in the Parisian distribution system were also analyzed based on statistics and econometrics approaches [25]. For Romania, only a few studies provide results on drinking water treatment [13,26,27].

To summarize, most results on the chlorine concentration series in water distribution systems use differential equations and a few other methods, such as artificial intelligence. Despite the last period, econometrics and hybrid methods proved their efficiency for modeling and forecast time series in different research fields, like economics [28,29,30], signal analysis [31], hydro-meteorology, environmental pollution [32,33,34,35], and pharmaceutics [36], they were less utilized in modeling the chlorine series at the outlet of the water treatment plants and in the water distribution systems.

In the above context, this article proposes alternative models (econometrics not based on differential equations) for the free chlorine residual concentrations series collected in the water treatment plant Palas (Constanta, Romania) from January 2013 to December 2018. It also emphasizes the possibility of using them for the forecast. The proposed approaches are univariate, not multivariate, as in most of the above-cited literature. They do not require deep specific knowledge in the modeling field (as in the case of differential equations and artificial intelligence) and are easily understood and utilized. Another advantage is extending the research to an area less explored in Romania, for which only a limited number of studies were performed. The models are compared, and their weaknesses and advantages are highlighted.

## 2. Materials and Methods

### 2.1. Data Series and Statistical Analysis

**Figure 1:**
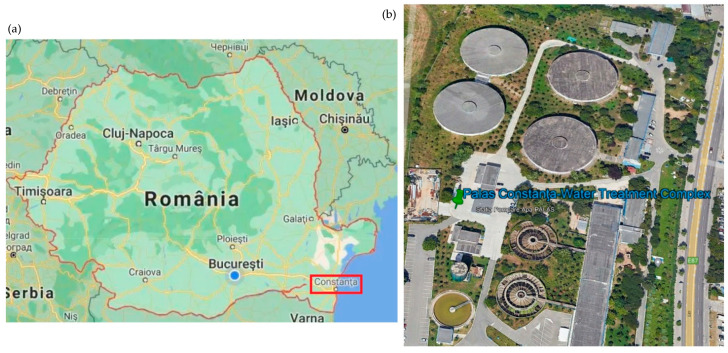
(**a**) Map of Romania; (**b**) the Palas Constanța water treatment complex (PCTC).

The Palas Constanţa treatment, storage, and pumping complex (PCTC) is located in the industrial area of Constanţa city on the Black Sea Littoral in Romania (Figure 1) and provides water to about 350,000 inhabitants.

The groundwater sources that feed the treatment plant are Cișmea I A, Cișmea I B, Cișmea I C, and Cișmea II. Cișmea I A + B+C are formed of 36 wells with depths from 50 to 120 m, except P35, with a depth of 300 m. They have a total supply capacity of 7657 m^3^/h. Cișmea II has 12 wells with depths between 90 and 150 m and a pumping capacity of 1940 m^3^/h. The Galeșu surface water source, with 13,050 m^3^/h catching capacity, is situated along the banks of Poarta Alba–Midia (on the Channel Danube–Black Sea). It has five intakes equipped with metal sieves for retaining the suspended particles.

This source was created to cope with the high water consumption during the summer and supplement Constanța city’s water supply when necessary. The water quality is good even before its treatment, according to [35,37]. After the treatment, the water must satisfy the Directives of the Council of the European Communities [38,39] and the Water Framework Directive [40]. The PCTC stores the water, which is distributed to Constanța and the Littoral water supply system. According to [41], in 2020, the total amount of water supplied to the inhabitants of Constanta was 42,150 m^3^ per day.

**Figure 2:**
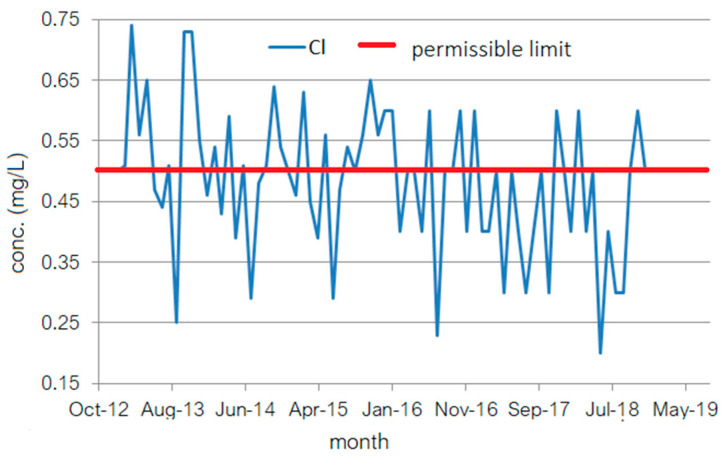
The monthly series of free chlorine residuals from January 2013 to December 2018.

Generally, for the drinking water distribution networks, there is a risk of insufficient drinking water distributed to consumers caused by phenomena such as the clogging of water sources or the lowering of the surface water level due to drought and lack of precipitation [42,43]. To avoid such situations, there are four water storage stations in Constanța, each of 20,000 m^3^, one of 6.000 m^3^, and another of 10,000 m^3^. The Caragea Dermen groundwater source can also be accessed. It is formed by 18 wells with depths between 35 and 90 m and has a supply capacity of 3.549 m^3^/h. The water from different sources undergoes different chlorination processes. Only after chlorination are the streams of water mixed and introduced into the distribution network. The studied data series (Figure 2) is formed of the monthly free chlorine residual concentration collected at the outlet of PCTP during January 2013–December 2018.

### 2.2. Statistical Analysis

Basic statistics (mean, median, standard deviation—SD, variation coefficient—CV) were first computed for the monthly series. Then, the following hypotheses were tested: normality against the non-normality (by the Jarque-Bera [44], Shapiro–Wilk [45], and Anderson–Darling [46] tests), homoscedasticity against heteroskedasticity (by the Levene test) [47], the series stationarity vs. its nonstationarity in mean and variance (by the KPSS test) [48]. The null hypothesis that there is no time series trend was tested against the alternative that a monotonic trend exists via the Mann–Kendall and seasonal Mann–Kendall test [49,50,51]. When the null hypothesis is rejected, Sen’s procedure [52] can be used to determine the monotonic trend.

### 2.3. Mathematical Modeling

Since the preliminary statistical analysis revealed the series seasonality, different approaches have been adopted to model the data series.

In the first approach, the series $\left(y_{t}\right)$ was decomposed using an additive model, of which its components are the trend, the seasonal component, and the random variable. In this case, the steps were the following [53]:

- Determine the trend using the linear trend computed via Sen’s method;
- Calculate the detrended series by subtracting the trend from the data series;
- Determine the seasonal component;
- Determine the remainder (random or residual component) as the difference between the detrended series and the seasonal component.

In the multiplicative decomposition, the steps are similar, but the addition is replaced by multiplication and the subtraction by division in the second and fourth steps from the previous method.

In the second approach, the decomposition was conducted following a similar procedure, but the trend was determined using a moving average method of the 12th order.

The third approach was to use the Holt–Winters method, where the series was decomposed using Equations (1)–(4) in the additive model, with a seasonal period *p* = 12 as follows:

(1) $${\hat{y}}_{t+h}=a_{t}+hb_{t}+s_{t-11+\left(h-1\right)mod\;12},$$

with

(2) $$a_{t}=\alpha \left(y_{t}-s_{t-12}\right)+\left(1-\alpha \right)\left(a_{t-1}+b_{t-1}\right),$$

(3) $$b_{t}=\beta \left(a_{t}-a_{t-1}\right)+\left(1-\beta \right)b_{t-1},$$

(4) $$s_{t}=\gamma \left(y_{t}-a_{t}\right)+\left(1-\gamma \right)s_{t-12},$$

In the multiplicative model, the equations are (5)–(8), which are expressed as follows:

(5) $${\hat{y}}_{t+h}={(a}_{t}+hb_{t})s_{t-11+\left(h-1\right)mod\;12},$$

where

(6) $$a_{t}=\alpha y_{t}/s_{t-12}+\left(1-\alpha \right)\left(a_{t-1}+b_{t-1}\right),$$

(7) $$b_{t}=\beta \left(a_{t}-a_{t-1}\right)+\left(1-\beta \right)b_{t-1},$$

(8) $$s_{t}=\gamma y_{t}/a_{t}+\left(1-\gamma \right)s_{t-12},$$

in the hypothesis that $a_{t}$ and $s_{t-12}$ are not zero.

In (1)–(8), α, β, γ are smoothing parameters that must be determined for the level, $a_{t},$ trend, $b_{t},$ and seasonal component, $s_{t},$ respectively [54].

The fourth proposed model is a Seasonal Autoregressive Integrated Moving Average model, SARIMA. An ARIMA (*p*,*d*,*q*) process ($x_{t}$) with a constant is defined by the following:

(9) $$\phi \left(L\right){\left(1-L\right)}^{d}y_{t}=c+\theta (L)\varepsilon_{t},$$

where *L* is the backward operator and

(10) $$\phi \left(L\right)y_{t}=\left(1-\sum_{i=1}^{p}\phi_{i}L^{i}\right)y_{t},$$

(11) $$\theta \left(L\right)\varepsilon_{t}=\left(1+\sum_{i=1}^{q}\theta_{i}L^{i}\right)\varepsilon_{t},$$

where

(12) $$y_{t}=x_{t}-x_{t-d}.$$

*p* and *q* are the numbers of autoregressive and moving average terms, respectively, *d* is the differentiation degree, and ${(\varepsilon }_{t})$ is white noise.

A SARIMA (*p*,*d*,*q*) × ${\left(P,D,Q\right)}_{m}$ (seasonal ARIMA model) is expressed as the following equation:

(13) $$\phi \left(L\right){\Phi (L^{m}){\left(1-L\right)}^{d}{\left(1-L^{m}\right)}^{D}y}_{t}=\theta \left(L\right)\Theta (L^{m})\varepsilon_{t},$$

where

(14) $$\Phi \left(L\right)y_{t}=\left(1-\sum_{i=1}^{P}\Phi_{i}L^{i}\right)y_{t},$$

(15) $$\Theta \left(L\right)\varepsilon_{t}=\left(1+\sum_{i=1}^{Q}\Theta_{i}L^{i}\right)\varepsilon_{t},$$

*m*, *D*, *P*, and *Q* represent the number of seasonal periods, the seasonal differencing, autoregressive, and seasonal moving average terms, respectively [55].

The residual independence was tested using the Box–Ljung test [56].

In all cases, apart from the residuals‘ analysis (normality, homoscedasticity, and randomness), the mean absolute deviation (MAD), mean standard deviation (MSD), and mean absolute percentage error (MAPE) were also computed to assess the models’ quality. Comparisons of the models, their advantages, and drawbacks are finally discussed.

The MINITAB 17, trial version (https://www.minitab.com/en-us/products/minitab/, accessed on 15 June 2023) and the R software, v.4.3.1 (https://www.r-project.org/, accessed on 15 June 2023) were utilized for testing the statistical hypotheses and mathematical modeling.

## 3. Results and Discussion

### 3.1. Results of the Statistical Analysis

The basic statistics of the data series are as follows: minimum = 0.200, maximum = 0.7400, mean = 0.4835, median =0.5000, standard deviation (SD) = 0.1181, coefficient of variance (CV%) = 24.42, skewness = −0.22, and kurtosis = −0.07. Thus, there is a small variation in the series values, and the distribution is left skewed.

Based on the above results, the computed value of the Jarque–Bera statistics was 0.4384, indicating that the normality hypothesis cannot be rejected at a significance level of 0.05. A similar result was obtained by applying the Shapiro–Wilk test. The *p*-value computed in the Levene test is 0.582 > 0.05, so the homoscedasticity hypothesis cannot be rejected. The statistics of the KPSS test for level (trend) stationarity is 0.59209 (0.03532), and the *p*-value is 0.02336 (0.1). So, the hypothesis of the level stationary is rejected, and that of the trend stationarity cannot be rejected at the significance level of 0.05. The Mann–Kendall test and its seasonal version rejected the null hypothesis. Therefore, based on Sen’s procedure, a linear trend, with the following Equation (16) can be fitted:

(16) $$Y_{t}=-0.001429(t-1)+0.542143,$$

where $Y_{t}$ is the concentration in the month t.

### 3.2. Models

**Figure 3:**
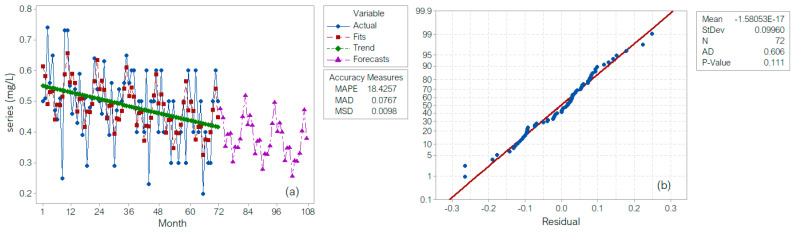
(**a**) Time series decomposition plot for the studied series. DECA; (**b**) the Q-Q plot of the random component. Mean is the average of the residual component’s values, StDev is the standard deviation of the residual component’s values, N is the number of the values, AD is the value of the Anderson–Darling statistics from the Anderson–Darling applied to the residual component, and P-value is the *p*-value computed in the Anderson–Darling test on the residual component.

When using the first approach, the series decomposition via the additive model (denoted as DECA) is presented in Figure 3a. The recorded (Actual) and the computed (Fitted) values are represented in blue and brown, respectively, and the trend is in green. The violet curve represents the series forecast for the next 48 months. The residuals are normally distributed, according to the Q-Q plot (Figure 3b) and the results of the Shapiro–Wilk test. They are homoscedastic (the *p*-value of the Levene test is 0.582 > 0.05) and autocorrelated (the first-order correlation coefficient is −0.3195).

**Figure 4:**
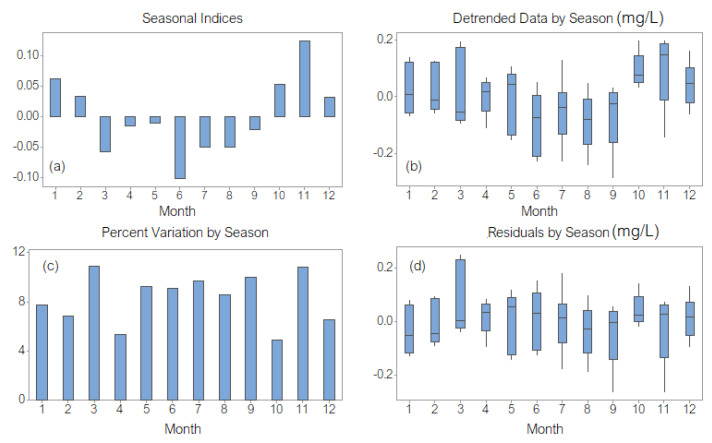
DECA: (**a**) Seasonal indices (1 corresponds to January and 12 to December); (**b**) Detrended data by season; (**c**) Percent variation by season; (**d**) Residuals by season.

The highest seasonal index corresponds to November, and the lowest to June (Figure 4a). The highest variations of the detrended series (Figure 4b) are those from November and March and the lowest from October.

The highest percentage variations per season (Figure 4c) were in March and November. The highest variation in the residual component (therefore, the worst fitted value) was in March, and the lowest one was in October (Figure 4d).

**Figure 5:**
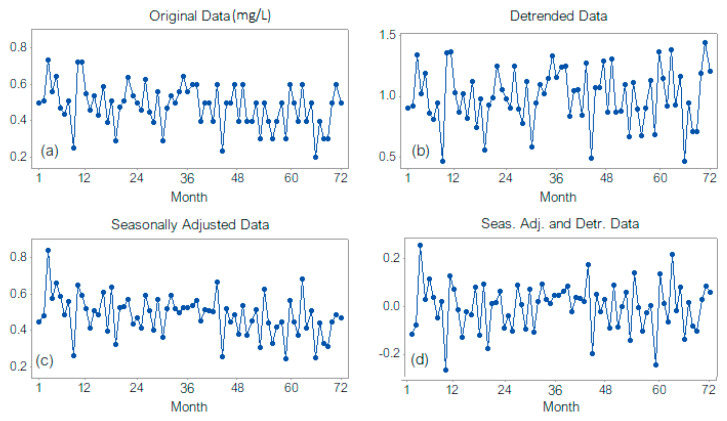
DECM: (**a**) Original Data, (**b**) Detrended series, (**c**) Seasonally adjusted series, (**d**) Seasonally adjusted and detrended series (residual component).

A similar behavior is noticed in the case of the multiplicative decomposition model (denoted in the following as DECM). Figure 5 shows the original series, the detrended one, the seasonally adjusted series, and the residual one.

Removing the trend from the initial series increases the series range. The seasonally adjusted series presents a lower variance than the original one, indicating that seasonality is a significant component of the series. The multiplicative decomposition model with a linear trend is slightly worse than the additive one since the mean absolute deviation (MAD) of 0.0773, mean standard deviation (MSD) of 0.0114, and mean absolute percentage error (MAPE) of 18.642 are higher than those in the additive model (0.0767, 0.0098, and 18.4257, respectively). Still, the models do not provide significant differences between the seasonal components, percent variation per season, or residuals per season. The hypotheses of the residuals series normality and homoscedasticity could not be rejected, but the randomness could. Therefore, one should look for a model with uncorrelated residuals to avoid the errors’ propagation.

**Figure 6:**
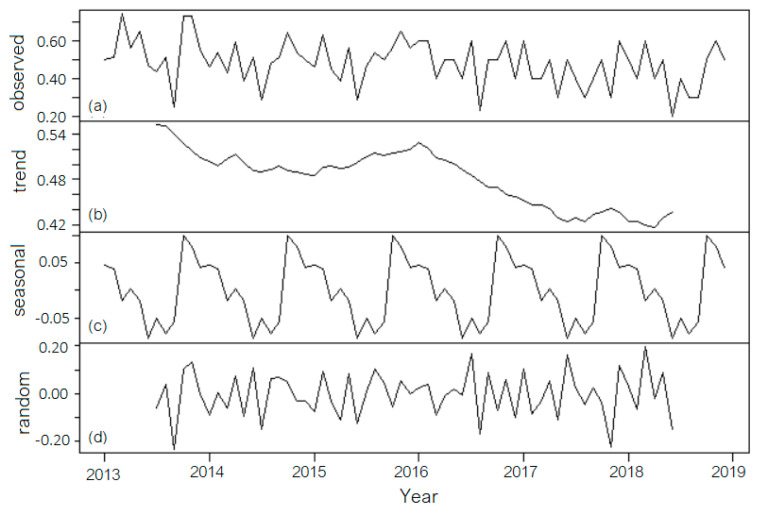
MAA12: (**a**) The initial series. (Observed is the default name given to it by the software); (**b**) Trend; (**c**) Seasonal component; (**d**) Random component.

In the second approach (decomposition with a 12th-order moving average trend), the best model was the additive one (denoted as MAA12). Figure 6 shows the initial series (observed), its trend, the seasonal, and the random component (residual). Due to the moving average computation, the trend is not linear or monotonically decreasing.

The seasonal indices are, respectively, Jan = 0.04668, Feb = 0.03876, Mar = −0.01790, Apr = 0.00360, May = −0.01940, June = −0.08790, July = −0.05149, Aug = −0.078569, Sept = −0.05648, Oct = 0.10001, Nov = 0.08059, and Dec = 0.04210. In this case, the highest values of the seasonal component are recorded in October, followed by November, and the lowest in June. The highest seasonal values are correlated to the higher chlorination necessity (in November and December) after the high season and the precipitation absence in summer (to maintain the quality of the drinking water), respectively, to the lowest chlorination necessity in June after the spring season and the high precipitation period.

**Figure 7:**
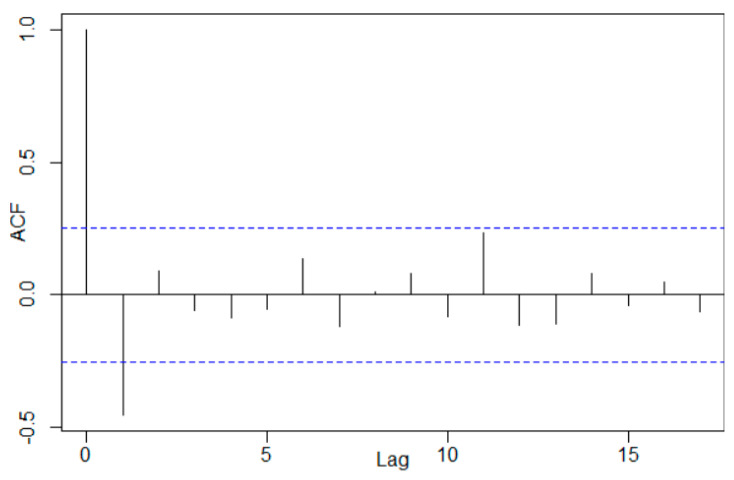
The random component’s correlogram in MAA12. The blue dotted line represents the limits of the confidence interval at a 95% confidence level.

The random component’s analysis provides a *p*-value of 0.9195 in the Shapiro–Wilk test, so the normality hypothesis cannot be rejected. The correlogram (Figure 7) shows again a first-order autocorrelation of the random component’s values.

The hypothesis of the random component’s homoscedasticity could not be rejected. For all statistical tests, the significance level was kept at 0.05. In MAA12, which is better than the multiplicative model with a 12th-order moving average trend (denoted as MAM12), MAD = 0.07601, MSD = 0.00870, and MAPE = 18.6546. In terms of MSD and MAD, the MAA12 is the best, while with respect to MAPE, the best is DECA. In both situations, a first-order autocorrelation of the residual series is present, so a third approach, the Holt–Winters method, was proposed to describe the series evolution.

**Figure 8:**
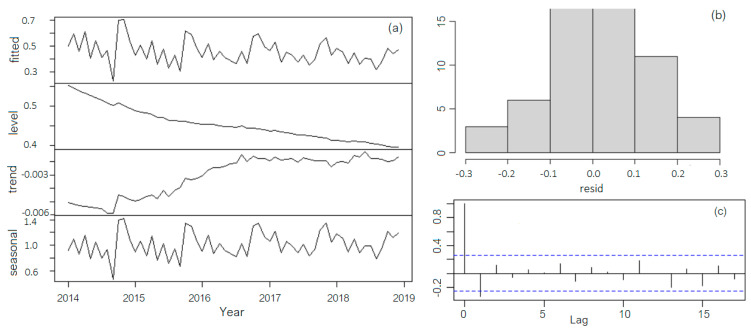
(**a**) Holt–Winters multiplicative model; (**b**) Residuals’ histogram; (**c**) Residuals correlogram.

Figure 8a provides the series decomposition using the multiplicative Holt–Winters method (denoted as MHW). The smoothing parameters are *α* = 0.04697, β = 0.07233, and γ = 0.43818, and the initial parameters and seasonality indices are *a* = 0.38112, b=−0.00191, $s_{1}$ = 0.10025, $s_{2}$ = 0.03342, $s_{3}$ = 0.06512, $s_{4}$ = 0.03010, $s_{5}$ = 0.01835, $s_{6}=$ −0.0806, $s_{7}=$ 0.00810, $s_{8}=$ −0.09001, $s_{9}=$ −0.04201, $s_{10}=$0.11143, $s_{11}=$ 0.12542, and $s_{12}=$ 0.11005.

In MHW, the level decreases in time, the trend increases (but not monotonically), and the seasonal component is not constant, according to the regression Equation (4) (or (8) in the multiplicative model).

Adding up the values of the level with the corresponding ones of the trend will result in a decreasing series of values (a decreasing trend in the first approach). Similar results were obtained using the multiplicative Holt–Winters method.

The level compound’s shape in MHW is concordant with the time series non-stationarity in level. Among the seasonal components, the highest values are recorded in November and October, followed by December. The seasonal values of the chlorine introduced in water in the treatment station after the high season are higher than in other periods (do not forget that the treatment plant is situated on the Black Sea Littoral in a tourist area, and during summer, the pollution is higher than in the rest of the year) and depends as well on the precipitation record during summer (that can carry the pollutants affecting the source water quality). In the additive Holt–Winters model (denoted as AHW), MAD = 0.0803, MSD = 0.0130, and MAPE = 18.8673, whereas in MHW, the corresponding values are MAD = 0.0772, MSD = 0.0118, and MAPE = 18.2619.

The tests on residuals did not reject their normality (see the histogram in Figure 8b) and homoscedasticity, but the randomness (see the correlogram in Figure 8c).

**Figure 9:**
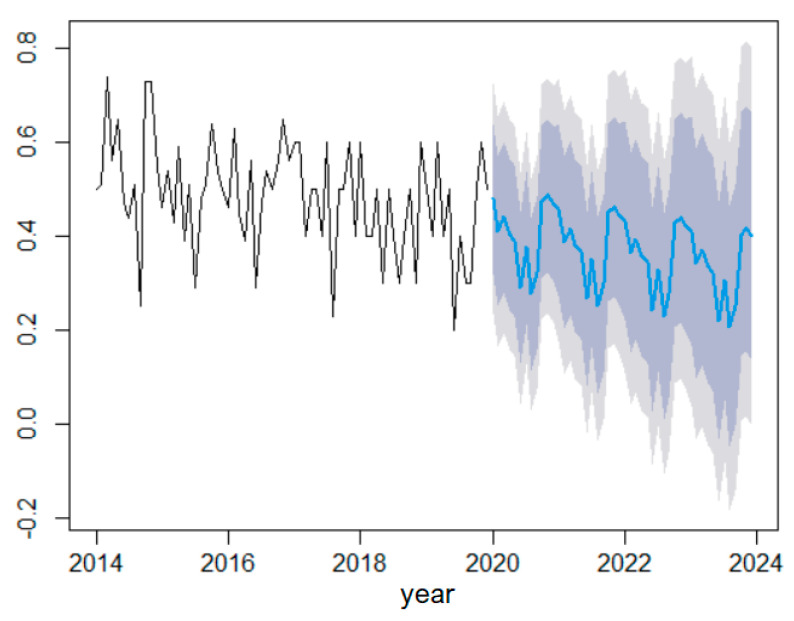
Forecast with the MHW model. The black curve is the series, the blue one is the forecast and the grey backgrounds are the confidence intervals at 95% and 99%, respectively.

Figure 9 illustrates the MHW model’s forecast for the next 48 months.

The series values are represented in blue, and the confidence intervals at 99% and 95% confidence levels are represented in two nuances of grey. The shape of the forecast curves is similar to that of the data series, confirming the modeling quality.

The advantage of this approach is that the level is considered, and the seasonal indices are updated at each step of the algorithm. The first two models incorporate the level and trend into a single component (trend), which does not reflect the series variation from the base.

**Figure 10:**
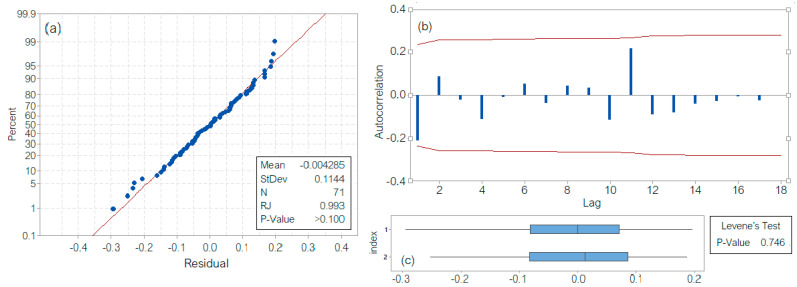
SARIMA model. Residual series analysis (**a**) Results of the Shapiro–Wilk test; (**b**) Correlogram; (**c**) Results of the Levene test.

The last model is of SARIMA(0,1,1)(0,1,1)_12_ type. For its validation, the residuals’ series analysis was performed. The Shapiro–Wilk test indicates that the hypothesis that the series in Gaussian cannot be rejected (*p*-value > 0.100 > 0.05; Figure 10a), the correlogram (Figure 10b) indicates the correlation absence, and the Levene test (Figure 10c) rejected the heteroskedasticity hypothesis. The *p*-value associated with the Box–Ljung test is *p* = 0.1137, indicating that the hypothesis of residuals’ series independence cannot be rejected. Moreover, MAD = 0.0695, MSD = 0.00868, and MAPE = 16.5426, showing that the SARIMA performs best among all the proposed models.

**Figure 11:**
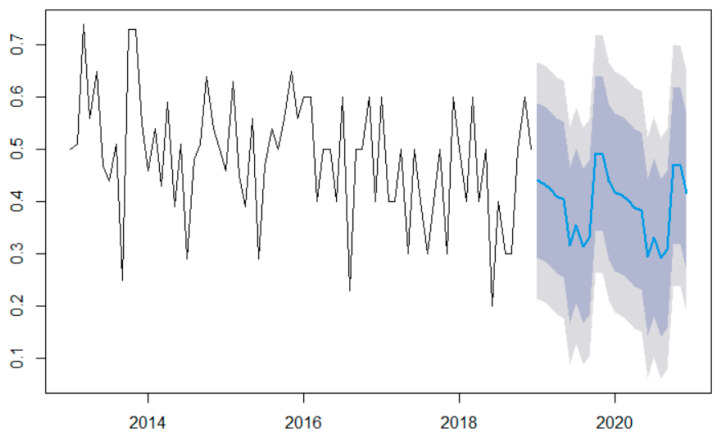
Forecast based on the SARIMA model. The black curve is the series, the blue one is the forecast and the grey backgrounds are the confidence intervals at 95% and 99%, respectively.

Figure 11 presents the series forecast based on the built SARIMA model in the blue curve and the confidence intervals at 99% and 95% confidence levels.

**Figure 12:**
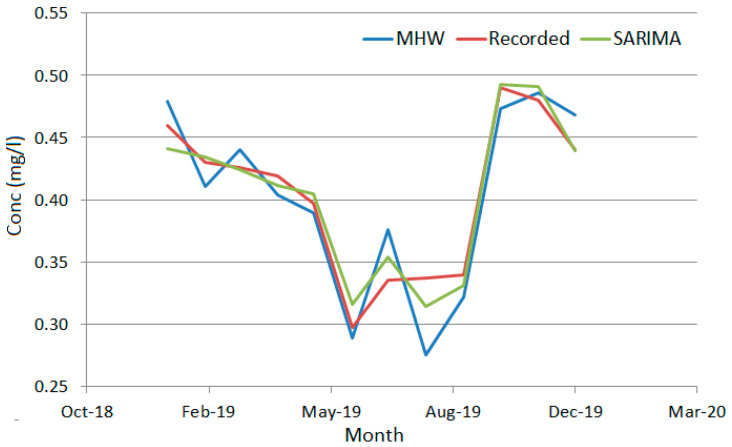
The series recorded in 2019 and the forecast based on MHW and SARIMA models.

To emphasize the performances of the forecast obtained using the MHW and SARIMA, their output was compared with the series values in recorded 2019 (that were not used for modeling). Figure 12 shows that the predicted values obtained using SARIMA are closer to the recorded values via comparison to MHW. The worst forecast was obtained for July and the best one for December.

The goodness of fit indicators for SARIMA (MHW) are MAD = 0.01039 (0.02118), MSD = 0.00016 (0.00068), and MAPE = 2.8181 (5.5738), showing that the SARIMA model is better than MHW.

All the approaches gave good results in modeling the free residual chlorine series, but the best (and more complex one) is the SARIMA(0,1,1)(0,1,1)_12_.

As mentioned, the chlorine quantity decreases during the disinfection processes due to the reactions with different substances. Keeping its concentration within optimal limits can be done if this parameter is monitored over time. Traditionally, process-based models to forecast chlorine decay use generally first-order equations [18,23,57]. To build such models, advanced knowledge of the phenomena that appear in the pipes, and accurate and sufficient data on some water parameters in the distribution system are necessary (the last must be experimentally obtained). Often, the coefficients in such models depend on the loading conditions and are not practical for modeling purposes [57]. Therefore, other approaches are required [17,21].

The second approach involves utilizing data-driven statistical models; this means that the forecast of residual chlorine utilizes relationships between the response variable and some regressors. If the experimental data on some variables are difficult to obtain, imprecise, or unavailable, the data-driven models are excellent alternatives to the process-based models [58]. In such models, the knowledge of the processes from the system is less important [14]. Their main advantage is that a deep knowledge of the mathematics and chemistry laws governing chlorine behavior is not necessary [59]. Among the data-driven statistical methods, we mention the linear autoregressive models to predict chlorine concentration and its decay in distribution systems and storage [14,60,61]. This present study falls into this category. Based on our best knowledge, it proposed four models that were first employed for modeling free chlorine monthly series. Therefore, comparisons with the results of similar studies conducted on different series cannot be performed.

## 4. Conclusions

This article proposed four alternative approaches for modeling monthly free chlorine residual concentration series from PCTP using decomposition, Holt–Winters, and SARIMA models. The novelty of this approach is the use of univariate econometric models in engineering and extending the results of other studies on the water treatment plant in Romania (that previously presented only basic statistical analysis or models of chlorine decay).

In the first approach, the trend was built using a nonparametric Sen’s method, which has the advantage that no other restrictions are to be satisfied by the parameters of the linear trend. Another advantage of this method is its simplicity. The second method has the advantage that it can be applied even in a situation when the hypothesis that a monotonic trend exists is rejected. Nevertheless, the twelve values of the series cannot be estimated. In the Holt–Winters method, the seasonality factors and the trend are updated at each step, which gives a more realistic picture of the evolution of each component compared to the classical decomposition. While the first two approaches are simpler, the third one includes a fourth component, the level, as a base from which the series vary. The Holt–Winters model is in concordance with the stationary test results. The SARIMA(0,1,1)(0,1,1)_12_ model is more complex since it involves the first-order differentiation of the series and its seasonal components (to reach its stationarity), and considering the innovation process (by the presence of the moving average, one for both series and seasonality). While the last methodology provides the most accurate results, all the others may be used for modeling and forecast given the easiness and availability of their implementation in MINITAB and R.

In Romania, the studies in the above field are either experimental, present basic statistics of some water parameters series (without correlations to each other) or use the first-order chlorine decay model. Therefore, this article completes the very sparse research in the field. Since the chlorine concentration is regularly monitored, and exceeding the limits imposed by regulation may give birth to protests from the residents that acknowledge the smell and taste of the drinking water, the amount of chlorine must be dosed taking into account the input water quality, resulting from the analyses of chlorine concentrations and the necessity to conform the Romanian regulations.

Despite their performances, the models presented here should be used only for short-time prediction without updating the input given a decreasing trend from the level from which the series’ values vary. Updating the input of the models is recommended for improving the forecast. Automating the chlorine concentration monitoring will result in a better dosage and forecast.

Another note is that the models do not include the risk factors and the solution for the situation when the water quality decreases. Therefore, in a future study, these aspects will be considered because there is a need to constantly monitor the water resources and the quality in the water treatment process and to intervene to maintain it.

## Data Availability

Data will be available upon request from the authors.
